# Epidemiological study on equine coccidiosis in North and Northeast of Iran

**DOI:** 10.1002/vms3.1197

**Published:** 2023-07-04

**Authors:** Faezeh Faghihzadeh Gorji, Soheil Sadr, Hassan Borji

**Affiliations:** ^1^ Department of Pathobiology Faculty of Veterinary Medicine Ferdowsi University of Mashhad Mashhad Iran; ^2^ Department of Clinical Sciences Faculty of Veterinary Medicine Ferdowsi University of Mashhad Mashhad Iran

**Keywords:** Coccidiosis, *Eimeria*, epidemiological study, horse, Iran, North, Northeast

## Abstract

**Background:**

*Eimeria* is a genus of protozoan parasites that infect many animal species, including horses. We conducted a cross‐sectional study of indigenous breeds of horses from the North and Northeast of Iran to establish the prevalence and distribution of *Eimeria* species.

**Material and methods:**

Using standard coprological techniques, 340 faecal samples from randomly selected horses (141 from North Iran and 199 from Northeast Iran) were examined for *Eimeria* oocyst.

**Results:**

Out of 340 samples, only three from north Iran were positive for coccidiosis. Infections occurred by *Eimeria leuckarti*. The mean intensity of oocyst output (3–38 o.p.g.) was very low. No clinical signs of gastrointestinal disorders were noticed in horses during this study.

**Conclusion:**

In conclusion, the results of this study suggest that the prevalence of *Eimeria* species causing coccidiosis in indigenous breeds of horses from the North and Northeast of Iran is relatively low. These findings provide valuable insights into the health status of indigenous horses in Iran and may help guide future efforts to promote their welfare and productivity.

## Graphical abstract

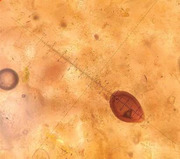

## 1 INTRODUCTION

Coccidiosis in equids is caused by protozoa of the genera *Eimeria* that infect the intestinal tract (Sazmand et al., 2020). Three species of *Eimeria* can infect horses; *E. uninugulata*, *E. solipedum* and *E. leuckarti* (Dubey & Bauer, 2018). Although horses characterized three species of *Eimeria*, only *E. leuckarti* is a valid species infecting equids, including horses, donkeys and zebra (De Souza et al., 2009). There is a prepatent period of 16–35 days for protozoal infection. As a result of consuming contaminated water or food containing sporulated oocysts, infection occurs (Fletcher et al., 2012). Sporozoites emerge from the oocysts after exposure to bile in the small intestine (Dubey et al., 2019). The oocysts shed as the final product of the sexual reproduction cycle infect new hosts through their faeces (Tyrnenopoulou et al., 2021). There is a greater prevalence of infections in foals aged 30–125 days compared to adults (Bianchi et al., 2019; Ola‐Fadunsin et al., 2019).

Some cases of *Eimeria* are located throughout the villus but typically in enterocytes displaced in the lamina propria (Yun et al., 2000). Immunohistochemistry studies have proven that the parasitized host cell is an enterocyte, not a vascular or mesenchymal cell (Bundina & Khrustalev, 2016). There may be erosions or ulcers on the villi, which may be atrophic (Hirayama et al., 2002). An eosinophilic infiltration is often observed with this infection; however, it is unclear whether it is specific to *E. leuckarti* or whether it is part of a helminth infection that is concomitant with this infection (Gülegen et al., 2016). In addition, there are also cases of haemorrhagic lesions accompanied by mild or severe mononuclear cell infiltrations.

In addition to being a universal disease, coccidiosis is most commonly seen in horses kept in small, oocyst‐contaminated areas. There are few reports on eimeriosis in equids in Iran, with infection rates of 0.5%–57.14% in horses and 7.7% in donkeys (Sazmand et al., 2020; Tavassoli et al., 2010). However, no comprehensive study explores these parasites due to inadequate research in the North and Northeast of Iran. Hence, the current study aimed to determine the prevalence of *Eimeria* in equines in the study area, identifying the most common species and the intensity of faecal oocyst output.

## 2 METHODS AND MATERIALS

As faecal samples were collected from the rectum of the animals for which animals were handled, the study procedure has been approved by the ethical committee of the Animal Welfare Committee Ferdowsi University of Mashhad (approval ID:IR.UM.REC.1400.12656860).

### 2.1 Study area

The study was conducted from October 2021 to July 2022 in two different climatic areas in Iran. The first region was Mashhad, the capital city of Khorasan Razavi in Northeast Iran. Mashhad is located in Northeast Iran, geographically located at 36°208′ north latitude and 59°358′ east longitude. Mashhad lies in the altitudinal range of 995 m above sea level, and its weather condition is cold, covering an area of 328 km^2^. Mashhad features a cold semi‐arid climate with hot summers and cold winters. The city only sees about 250 mm (9.8 in.) of precipitation annually, some occasionally falling in snow.

The second region was Babol, one of the most important cities in the north of Iran, between the northern slopes of the Alborz coast of the Mountains and the southern Caspian Sea. The city is approximately 20 km south of the Caspian Sea on the west bank of the Babolrud River and receives abundant annual rainfall. Babol has a humid subtropical climate that borders on a Mediterranean climate.

### 2.2 Study animals and design

To determine *Eimeria* prevalence and potential risk factors, coprological examinations were conducted on 340 randomly selected indigenous breeds of horses. Of 340 stool samples, 199 were taken from Mashhad, and 141 were taken from Babol.

After the samples were collected from the selected site, they were transported to the parasitology laboratory for further examination and analysis. During transportation, care was taken to ensure that the samples were not exposed to extreme temperatures or other factors that could alter their composition. At the laboratory, the samples were stored under appropriate conditions until they were ready for analysis.

For the quantification of parasites, oocysts per gram (OPG) were quantified at 10× and 40× magnifications using a modified McMaster standard. This method involves preparing a slide with a known volume of the faecal sample and counting the number of eggs or oocysts under the microscope (Studzińska et al., 2008). The number of eggs or oocysts counted is then used to calculate the OPG, which estimates the number of parasites in the sample. Overall, the transportation, preparation and analysis of the collected samples were carried out with great care and attention to detail, ensuring that the results were accurate and reliable.

## 3 RESULTS

Out of the 340 samples collected from horses in the study, only three samples from north Iran were positive for coccidiosis. The *E. leuckarti* species caused the infections. The mean intensity of oocyst output was very low, ranging from 3 to 38 OPG of feces. This is a common observation in cases of *E. leuckarti* infections, where most infected animals show no apparent clinical signs. In this study, only one foal showed mild clinical signs of infection (Table 1).

**TABLE 1:** Infected horses information.

Horse no.	Age	Sex	Breed	History of anti‐parasitic treatments	Clinical signs
1	1	Male	Turkmen	No	Mild diarrhoea–anorexia–mild depression
2	1.5	Male	Caspian	No	Mild diarrhoea
3	2.5	Female	Caspian	No	No clinical signs

*Note*: A comprehensive overview of the infected horses and their associated details includes the age, clinical signs, history of anti‐parasitic treatments, sex and breed of the horses.

Our observation in this study revealed that only a few foals exhibited temporary diarrhoea, a common symptom of coccidiosis in horses. This suggests that the horses in the study may have developed immunity against the parasite, a typical response to repeated exposure to coccidian oocysts.

**FIGURE 1:**
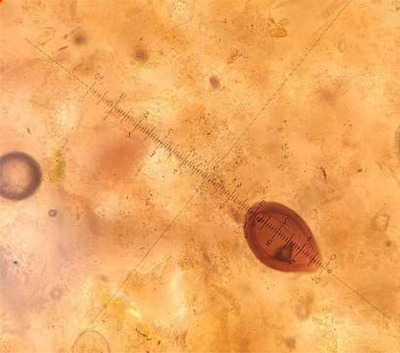
*Eimeria leuckarti* oocyst. The size of the *E. leuckarti* oocyst was estimated to be (75 ± 3.75 × 58 ± 2.9) μm 95% CI.

Based on the counting results, 20 eggs were counted from each of the 3 sick horses, making a total of 60 eggs. The estimated size of the *E. leuckarti* species was (75 ± 3.75 × 58 ± 2.9) μm 95% CI, which falls within the expected range for coccidian oocysts (Figure 1).

## 4 DISCUSSION

In this study, the protozoan *E. leuckarti* was observed in three cases of the studied horses from north Iran. Ahmadi et al. has reported four *E. leuckarti‐*infected samples in Shahrekord district horses (Ghahfarrokhi et al., 2014). According to the results of this study and the study conducted in the Shahrekord region, it can be concluded that *E. leuckarti* exists in Iranian horses with a low prevalence. There have been reports of *E. Leuckarti* parasites on horses in North America and other parts of the world, but they are ubiquitous protozoal parasites (Attia et al., 2018; Jenkins et al., 2020; Jota Baptista et al., 2021). As a result of a survey conducted on Kentucky horse farms, 41% of foals examined were found to have an oocyst, and 85% of farms had foals with an oocyst, according to the survey (Lyons & Tolliver, 2004; Lyons et al., 2006). Studzińska et al. (2008) reported 9.18% *E. leuckarti* in Poland, and Gülegen et al. (2016) reported 2.9% *E. leuckarti* in Turkey.

The protozoan parasites of the genus *Eimeria* multiply in the intestinal tract and cause tissue damage, with resulting interruption of feeding and digestive processes or nutrient absorption; dehydration; blood loss; and increased susceptibility to other disease agents (Blake et al., 2020; Gajadhar et al., 2015; Gutiérrez‐Expósito et al., 2017). The disease may be mild, resulting from ingesting a few oocysts, and may escape notice, or it may be severe due to the ingestion of millions of oocysts (Kompi et al., 2021). Like many parasitic diseases, coccidiosis is largely a disease of young animals because immunity quickly develops after exposure and gives protection against later disease outbreaks (Abbas et al., 2019).

Despite the lower prevalence of coccidian disease in horses, sporadic clinical cases and deaths are still possible, especially in young foals starting to wean (Mulwa et al., 2020). Compared to other animals, it is much less common for horses to be affected by clinical *E. leuckarti* than in other species. However, clinical cases and deaths occur occasionally, especially in foals just recently weaned from their mothers (Kalef, 2015). Regardless of the facility the horses are kept in, coccidiosis can strike any type of horses (dos Santos et al., 2014). Despite the fact that there is some doubt about the pathogenicity of *E*. *leuckarti* in horses, it has been described in foals and young horses to exhibit diarrhoea lasting several days and to develop an acute massive intestinal haemorrhage leading to rapid death from the disease (Kornaś et al., 2011; Marinković et al., 2013).

Furthermore, in current study, it is important to note that the absence of clinical signs does not necessarily indicate the absence of clinical impact. Equine coccidiosis may still have subtle effects on horse health and well‐being that have not yet been adequately investigated. Thus, researchers and practitioners must remain vigilant and consider the possibility of equine coccidiosis as a potential contributing factor in unexplained performance decline or health issues.

Based on the results of the present study, it can be concluded that some examined horses in northern Iran suffer from subclinical coccidiosis and are infected with different *Eimeria* species. Although none of the horses had clinical signs of coccidiosis, there was evidence of subclinical contamination with coccidiosis in the herd. Thus, more research needs to be carried out on this subject in local rural areas in Iran and pay greater attention to the molecular identification and characterization of the species of *Eimeria* found there. Based on these results, Iran can develop a strategy to control the disease based on relevant data that will provide a solid foundation.

## 5 CONCLUSION

Overall, the low prevalence and intensity of coccidiosis infections in the horses in this study and the absence of clinical signs indicate that the disease may not be a major concern in the horse population in the study area. However, further studies are needed to determine the extent of coccidiosis infections in other regions and populations of horses and to evaluate the potential impact of the disease on equine health and performance.

## AUTHOR CONTRIBUTIONS

*Conceptualization; supervision*: Hassan Borji. *Methodology, formal analysis and investigation; writing – review and editing*: all authors. *Writing – original draft preparation*: Soheil Sadr. *Funding acquisition*: self‐funding. All authors checked and approved the final version of the manuscript for publication in the present journal.

## CONFLICT OF INTEREST STATEMENT

The authors declare no conflicts of interest.

## ETHICS STATEMENT

All applicable international, national and/or institutional guidelines for the care and use of animals were followed. Ethical No. (approval ID:IR.UM.REC.1400.12656860).

### PEER REVIEW

The peer review history for this article is available at https://publons.com/publon/10.1002/vms3.1197.

## Data Availability

The datasets generated during and/or analysed during the current study are available from the corresponding author on reasonable request.
